# Postbiotic *Lactobacillus reuteri* LRE02 Enriched with Sodium Selenite Demonstrates Antidepressant- and Anxiolytic-Like Effects in Mice

**DOI:** 10.1007/s12035-026-06000-w

**Published:** 2026-06-19

**Authors:** Aline Silveira Gonçalves, Mariana Parron Paim, Narryman Pinto Zuge, Marcia Juciele da Rocha, Marcelo Heinemann Presa, Evelyn Mianes Besckow, Fábio Pereira Leivas Leite, Lucielli Savegnago, Fernanda Severo Sabedra Sousa, Fabiana Kommling Seixas, Tiago Veiras Collares, Gustavo Gohlke, Erico Marlon de Moraes Flores, Marcia Foster Mesko, Cristiani Folharini Bortolatto, Paloma Taborda Birmann, César Augusto Brüning

**Affiliations:** 1 Laboratory of Biochemistry and Molecular Neuropharmacology (LABIONEM), Graduate Program in Biochemistry and Bioprospecting (PPGBBio), Chemical, Pharmaceutical, and Food Sciences Center (CCQFA), Federal University of Pelotas (UFPel), Pelotas, RS 96010-900 Brazil; 2 Graduate Program in Biotechnology (PPGB), Center for Technological Development, Federal University of Pelotas, Pelotas, RS Brazil; 3 Graduate Program in Biotechnology (PPGB), Research Group On Neurobiotechnology - GPN, Center for Technological Development, Federal University of Pelotas, Pelotas, RS Brazil; 4 Graduate Program in Biotechnology (PPGB), Research Group On Cellular and Molecular Oncology - GPO, Center for Technological Development, Federal University of Pelotas, UFPel, Pelotas, RS Brazil; 5 Laboratory of Control of Contaminants in Biomaterials (LCCBio), Graduate Program in Biochemistry and Bioprospecting (PPGBBio), Chemical, Pharmaceutical, and Food Sciences Center (CCQFA), Federal University of Pelotas (UFPel), Pelotas, RS 96010-900 Brazil; 6 Department of Chemistry, Graduate Program in Chemistry (PPGQ), Federal University of Santa Maria, Santa Maria, RS 97105-900 Brazil

**Keywords:** Postbiotics, *Lactobacillus*, Selenium, Oxidative stress, Inflammation, Neuropsychiatric disorders

## Abstract

Postbiotics, including inactivated microorganisms and their bioactive components, have emerged as innovative candidates for microbiota-targeted therapies. Among them, *Lactobacillus reuteri*-derived postbiotics represent a promising and underexplored strategy for modulating host physiology without the risks associated with live bacteria. Selenium enrichment may further enhance these benefits by strengthening antioxidant defenses and regulating inflammation, two central mechanisms to microbiota–gut–brain axis communication. In this study, we investigated the antidepressant- and anxiolytic-like effects of *L. reuteri* LRE02 in three formulations: a live probiotic (Lr), an inactivated postbiotic (ILr), and a selenium-enriched postbiotic (ILr/Se) in male Swiss mice. Animals were assigned to five groups: vehicle, Lr, ILr, ILr/Se, and fluoxetine, and treated intragastrically for 14 days (300 μL, 10^9^ CFU/mL). Behavioral tests and biochemical analyses were subsequently performed. All *L. reuteri*–based treatments presented antidepressant-like effect and reduced corticosterone levels, indicating HPA axis modulation. The postbiotic formulations also improved oxidative stress parameters, reduced TNF-α and IL-6 across the prefrontal cortex, hippocampus, and small intestine, and restored IDO expression. Notably, ILr/Se exhibited anxiolytic-like effects and upregulated PI3K/Akt/mTOR signaling and NRF2 expression in the intestine, suggesting enhanced redox and neuroplasticity support. The key innovation of this work lies in demonstrating that inactivated and selenium-enriched *L. reuteri* postbiotics exert antidepressant-like and anxiolytic-like effects, offering advantages in stability, safety, and reproducibility. These findings highlight postbiotics, especially when combined with selenium, as promising candidates for future microbiota-based interventions targeting mental health.

## Introduction

The genus *Lactobacillus* was among the first probiotics to be identified and is widely used in food products and nutritional supplements due to its health-promoting properties [1]. Among these species, *Lactobacillus reuteri* stands out due to its abundance and extensive characterization, exhibiting biological functions shaped by host and environmental factors [2]. Its capacity to modulate the gut microbiota, produce antimicrobial molecules like reuterin, interact with epithelial and immune cells, and generate anti-inflammatory and antioxidant metabolites underlies its well-documented efficacy in gastrointestinal and systemic conditions [2–6]. Clinical evidence further shows that *L. reuteri*, particularly in multi-strain formulations, enhances immune activity and reduces oxidative and inflammatory biomarkers, reinforcing its relevance as a therapeutic microorganism [7].

Probiotics such as *Lactobacillus* and *Bifidobacterium* confer health benefits when consumed in adequate amounts, yet emerging evidence reveals that biological activity is not restricted to live cells [8, 9]. Inactivated microorganisms and their structural or metabolic components, postbiotics, retain functional bioactivity even after loss of viability, offering advantages such as greater stability, safety, and reduced risk of microbiota disruption [10–12]. These bioactive preparations act through metabolites and signaling molecules generated before inactivation, expanding the therapeutic potential of microbial-derived interventions beyond traditional probiotics [11–13].

The development of selenium-enriched postbiotics adds a further layer of biological relevance, since selenium is a key micronutrient involved in antioxidant defense, immune regulation, and redox homeostasis [14–16] and can shape gut microbial composition [17]. This strategy becomes particularly promising in the context of major depressive disorder and anxiety disorders, highly prevalent neuropsychiatric conditions whose incidence has risen sharply in recent years [18–20]. Growing evidence implicates the microbiota–gut–brain axis in the modulation of mood and emotional regulation, with microbial metabolites influencing neurochemical pathways, inflammation, and stress responses [21–24]. Notably, *L. reuteri* has been shown to alleviate depression- and anxiety-like behaviors in murine models, and its abundance is increased by antidepressants such as fluoxetine [25]. Moreover, combined supplementation of probiotics and selenium has demonstrated synergistic effects in reducing oxidative stress and inflammation [26, 27].

In this context, the present study investigates whether the sodium selenite-enriched *Lactobacillus reuteri* LRE02 postbiotic can modulate depression- and anxiety-like behaviors in mice, focusing on its effects on oxidative stress, neuroplasticity, and inflammatory pathways within the microbiota-gut-brain axis.

## Materials and Methods

### Preparation of *Lactobacillus reuteri* LRE02

The *Lactobacillus reuteri* LRE02 strain used in this study was obtained from Probiotical and is registered under DSM 23878 in the Deutsche Sammlung von Mikroorganismen und Zellkulturen (DSMZ) collection. The strain was cultured in Man Rogosa and Sharpe (MRS) medium and maintained in an orbital shaker at 37 °C until reaching 10^9^ colony-forming units (CFU)/ml, a concentration based on previous preclinical studies of depression and anxiety [27, 28]. The culture was then divided into three preparations: live microorganisms (Lr), a selenium-enriched postbiotic (ILr/Se), and a heat-inactivated postbiotic (ILr). To produce ILr/Se, sodium selenite (200 μg/ml) was added to the culture and incubated at 37 °C for 24 h, following established protocols [29, 30]. Thermal inactivation for ILr was performed by heating at 80 °C for 40 min. Bacterial viability was assessed using the colony-forming unit (CFU) method on MRS agar. For the ILr/Se group, plating at 3, 5, 7, and 24 h after selenite addition showed growth up to 7 h, with complete inactivation after 24 h of exposure to sodium selenite. ILr cultures were confirmed as inactive following heat treatment, and ILr/Se cultures were confirmed as inactive after sodium selenite addition. All preparations were stored at 4 °C until administration.

### Determination of Selenium in Bacterial Culture

Samples of the bacterial culture were dried in an incubator (Marconi, Brazil) at 60 °C for 5 h until a constant weight was achieved. The dried material was ground using a cryogenic mill (Cole-Parmer, USA) and digested in a microwave system (Ethos Easy, Milestone, Italy) with a rotor accommodating forty-four 100 mL modified Teflon® flasks. Approximately 50 mg of each sample (in duplicate) was digested with 6 mL of distilled nitric acid. The microwave program consisted of (i) ramping to 200 °C over 20 min, (ii) maintaining 200 °C for 20 min, and (iii) cooling to 50 °C. After digestion, the final volume was adjusted to 20 mL with ultrapure water. Selenium content was determined by inductively coupled plasma mass spectrometry (ICP-MS, Elan DRC II, Perkin Elmer, Canada) using the ^82Se isotope, within a linear range of 0.5–10 μg L⁻^1^.

### Animals

This study was approved by the Animal Ethics Committee of the Federal University of Pelotas (CEUA-UFPel; protocol number 035551/2023-47). Healthy male Swiss mice, obtained from the central animal facility of UFPel, weighing 25–30 g, were housed five per cage under standard conditions in the central animal facility, with a 12 h/12 h light–dark cycle, controlled temperature (22 ± 1 °C), and ad libitum access to food and water. All procedures were conducted to minimize animal suffering in accordance with NIH guidelines for the care and use of laboratory animals (NIH Publication No. 8023, revised 1978) and the ARRIVE Guidelines for Reporting Animal Research.

### Experimental Design

**Fig. 1:**
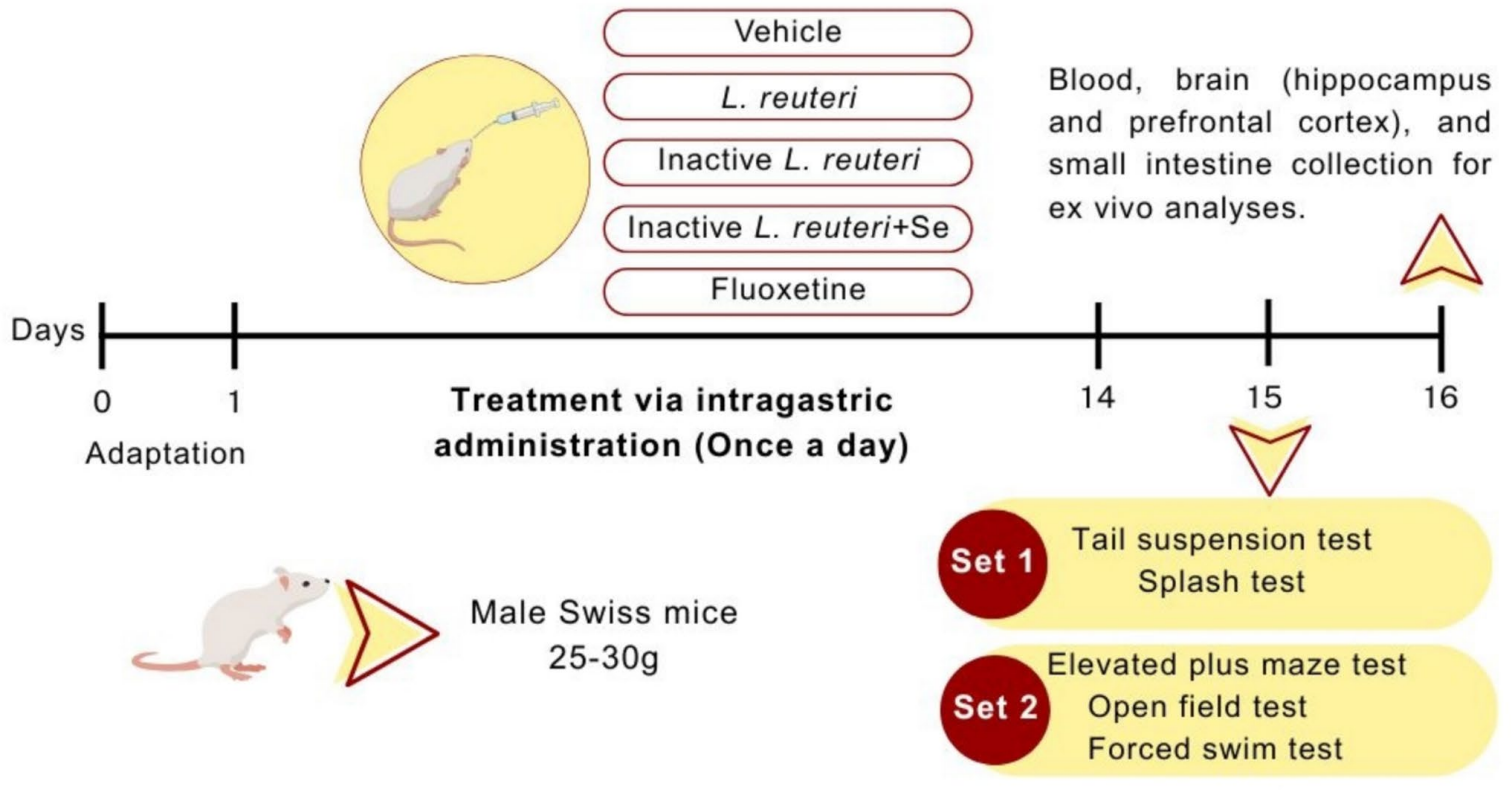
Experimental design

The animals were randomly assigned to the following groups (*N* = 7 per group). Vehicle (V), receiving 300 μL of MRS; *Lactobacillus reuteri* (Lr), receiving 300 μL (10^9^ CFU/mL) of the probiotic; inactive *L. reuteri* (ILr), receiving 300 μL (10^9^ CFU/mL) of the postbiotic; and inactive *L. reuteri* with selenium (ILr/Se), receiving 300 μL (10^9^ CFU/mL; 60 µg of selenium) of the selenium-enriched probiotic. Treatments containing inactivated bacteria were plated before and after inactivation to confirm effectiveness. The positive control group received fluoxetine (Flx) at a dose of 5 mg/kg body weight, as previously demonstrated to be effective in earlier studies [31–33]. The study was conducted in two experimental sets to optimize procedures. To reduce stress during behavioral assessments, tests associated with behavioral despair were conducted in separate sets, ensuring that no individual animal underwent both the tail suspension and forced swim tests. Furthermore, to maintain randomization, animals from different experimental groups were tested in an alternating sequence within each session. After a 1-day adaptation, animals were treated once daily via intragastric administration for 14 days. In set 1, on day 15, the animals underwent the tail suspension test and subsequently the splash test. In set 2, the elevated plus maze, open field test, and forced swim test were performed in that order. In both sets, euthanasia and tissue collection were conducted on day 16, with samples stored at −80 °C for further analyses. The experimental design is illustrated in Fig. 1.

### Behavioral Tests

#### Open Field Test (OFT)

The OFT was performed to exclude potential locomotor or exploratory impairments induced by the treatments [34]. Mice were placed in the center of a wooden box (30 × 30 × 15 cm) divided into nine equal quadrants. Locomotor activity (number of quadrant crossings) and exploratory activity (rearings on the hind paws) were recorded for 4 min.

#### Tail Suspension Test (TST)

Each mouse was suspended by the tail on a wooden apparatus 50 cm above the floor. The total immobility time over 6 min was measured. Immobility, defined as the absence of escape-oriented behavior, is considered indicative of depressive-like behavior [35].

#### Splash Test (ST)

The ST evaluates motivational and self-care behavior [36, 37]. A 10% sucrose solution was sprayed on the dorsal coat of each mouse, eliciting instinctive grooming. The total time spent grooming was recorded over 5 min.

#### Forced Swimming Test (FST)

The FST assesses depressive-like behavior by measuring immobility as an absence of escape-directed behavior [38]. Mice were individually placed in a cylinder (10 cm diameter × 25 cm height) filled with 19 cm of water at 25 ± 1 °C. The total immobility time was recorded during a 6-min test.

#### Elevated Plus Maze Test (EPMT)

The EPMT evaluates anxiety-like behavior [39]. The apparatus consists of a cross-shaped wooden maze with two open arms (30 × 5 cm), two closed arms (30 × 5 × 15 cm), and a central platform (5 × 5 cm). Anxiety parameters measured were the number of entries into the open arms and the time spent there.

### Tissue Collection

The mice were anesthetized with isoflurane-soaked cotton, and blood was collected via cardiac puncture using a heparinized syringe. The blood was immediately centrifuged at 3600 × *g* for 10 min at 4 °C. Cervical dislocation was performed to confirm euthanasia, and the hippocampus (HC), prefrontal cortex (PFC), and small intestine (SI) were collected. Tissues were stored at −80 °C until further analysis. Although previous studies have reported neuroprotective effects of isoflurane [40, 41], this potential effect was evenly distributed across all groups, thereby minimizing any treatment-related bias.

### Biochemical Analysis

#### Evaluation of Plasma Corticosterone Concentration

Plasma corticosterone was quantified using a three-step assay. In the first step, the steroid hormone was extracted with the organic solvent chloroform. In the second step, the extract was treated with 0.1 M sodium hydroxide for purification. In the final step, the sample was reacted with a fluorescence reagent at room temperature for 2 h in the dark. Corticosterone levels were measured using a fluorometer (Excitation: 247 nm, Emission: 540 nm, beam: 10), and results were expressed as ng/mL of plasma [42].

#### Determination of Antioxidant Ferric Reducing Power (FRAP)

The assay is based on the reduction of ferric ions (Fe^3^⁺) to ferrous ions (Fe^2^⁺) under acidic conditions, forming a colored complex measurable by spectrophotometry [43]. The FRAP solution was prepared by mixing 38 mM sodium acetate, 20 mM FeCl₃, and 10 mM 2,4,6-tri(2-pyridyl)-s-triazine (TPTZ) in 40 mM HCl at a ratio of 10:1:1. The solution was added to tubes containing 25 μL of plasma, followed by incubation at 37 °C for 40 min in the dark. Absorbance was measured at 593 nm.

#### Thiobarbituric Acid Reactive Species (TBARS) Assay

Lipid peroxidation in the PFC, HC, and SI was assessed by malondialdehyde (MDA) formation using the thiobarbituric acid reactive substances (TBARS) assay. Tissue samples were homogenized in 50 mM Tris–HCl (1:10 w/v), centrifuged at 3600 × *g* for 10 min at 4 °C, and the supernatant collected for analysis. An aliquot of the supernatant was incubated with 8.1% sodium dodecyl sulfate (SDS), 0.8% thiobarbituric acid (TBA), and acetic acid/HCl (pH 3.4) at 95 °C for 2 h. TBARS levels were determined spectrophotometrically at 532 nm and expressed as nmol TBARS/mg protein [44].

#### Nitrate/Nitrite (NOx) Levels

Nitrite levels in the SI were determined spectrophotometrically at 540 nm, based on the reduction of nitrate to nitrite by vanadium chloride (VCl₃) [45]. Tissue samples were homogenized in 50 mM Tris–HCl (1:10 w/v), centrifuged at 3600 × *g* for 10 min at 4 °C, and the supernatant collected for analysis. Results were expressed as nmol NOx/mg protein.

### TNF-α Quantification by ELISA Kit

HC, PFC, and SI samples were homogenized in 50 mM Tris–HCl (1:10 w/v), centrifuged at 3600 × *g* for 10 min at 4 °C, and the supernatant collected for analysis. TNF-α levels were quantified using an ELISA kit (RAB0477, Sigma-Aldrich) according to the manufacturer’s protocol. Briefly, 100 µL of dilution buffer, standards, or samples were added to the wells and incubated for 2.5 h at room temperature with gentle shaking. After washing, 100 µL of biotinylated antibody was added and incubated for 1 h. The wells were washed again, followed by addition of 100 µL of streptavidin and a 45-min incubation. After a final wash, 100 µL of one-step TMB reagent was added and incubated for 30 min in the dark. The reaction was stopped with 50 µL of stop solution, and absorbance was measured at 450 nm. TNF-α concentrations were calculated using a standard curve (46.87–3000 pg/mL) and expressed as pg/mL.

### Real-Time Polymerase Chain Reaction Analysis

Total RNA was extracted from the HC, PFC, and SI using TRIzol, followed by quantification. cDNA synthesis was performed with the High-Capacity cDNA Reverse Transcription Kit according to the manufacturer’s instructions. qPCR amplification was carried out using GoTaq® qPCR Master Mix on a Stratagene Mx3005P real-time PCR system. Gene expression levels were normalized to GAPDH as the reference gene. The following genes were analyzed (Table 1).

**Table 1:** Primer sequences used in the real-time polymerase chain reaction analysis

Gene	Sequence 5′–3′
*GAPDH*	F: GGGTGAGGCCGGTGGTGCTGAG R: TGGGGGTAGGAACACGGAAGG
*AKT*	F: TGGGAGTAGACAAGGTACAACCC R: GGTGTCAGTCTCCGACGTG
*BDNF*	F: TAACGGCGGCAGACAAAAAGACT R: TGTCTCTATCCTTATGAATCACCAGCCAA
*IDO*	F: AATCAAAGCAATCCCCACTG R: AAAAACGTGTCTGGGTCCAC
*IL-6*	F: CCAGAAACCGCTATGAAG R: CACCAGCATCAGTCCCAAGA
*mTOR*	F: AGAAGGGTCTCCAAGGACGACT R: GCAGGACACAAAGGCAGCATTG
*NRF-2*	F: GACGCAGACCCTCTCTCTTGTC R: TAAGCGAACACCAAGCTCCT
*NRLP3*	F: GGTCCTCTTTACCATGTGCTTC R: AAGTCATGTGGCTGAAGCTGTA
*Synaptophysin*	F: TGT GTT TGC CTT CCT CTA CTC R: TCA GTG GCC ATC TTC ACA TC
*PI3K*	F: CTGGAGAGTTTAGAGGACG R: AAGCCACGCTTCAGCAGGAATC

### Protein Quantification

Protein concentration was determined using the Bradford assay with Coomassie Brilliant Blue and the S1 tissue supernatant. Absorbance was measured at 595 nm, and protein levels were calculated using a bovine serum albumin (BSA) standard curve. Samples were obtained from the HC, PFC, and SI. This assay was performed to ensure accurate measurement and normalization of TBARS and nitrite levels [46].

### Statistical Analysis

Data normality was assessed using the D’Agostino-Pearson test. The N of the experiments was validated by G*Power version 3.1.9.7 (Franz, Universitat Kiel, Germany). Statistical analyses were performed using one-way ANOVA followed by the Newman-Keuls post hoc test. Results are presented as mean ± standard error of the mean (SEM), and differences were considered statistically significant at *P* < 0.05. All analyses were conducted using GraphPad Prism 8.0 software.

## Results

### *L. reuteri* Enrichment with Sodium Selenite

The results obtained after sample drying, digestion, and selenium quantification by ICP-MS are presented in Table 2. As shown, *L. reuteri* enriched with sodium selenite exhibited a markedly higher selenium concentration compared to *L. reuteri* without supplementation.

**Table 2:** Selenium concentrations (μg ^g−1^) after decomposition in a microwave oven and determination by ICP-MS (mean ± standard deviation, *n* = 2)

Sample	Se concentration, μg ^g−1^
*L. reuteri* (no Se)	< 0.311*
*L. reuteri* (with Se)	14,580 ± 414

*LOQ obtained after determination by ICP-MS

### Effects of Lr, ILr, and ILr/Se on Locomotor and Exploratory Behavior of Mice

**Fig. 2:**
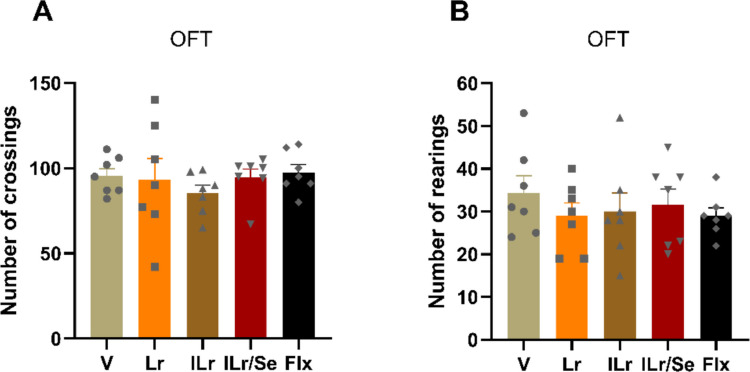
Behavioral assessment in the open field test. **A** Number of crossings and **B** number of rearings. Data were analyzed using one-way analysis of variance (ANOVA) followed by the Newman–Keuls post hoc test. *n* = 7 animals per group. V: vehicle; Lr: *L. reuteri*; ILr: inactive *L. reuteri*; ILr/Se: inactive *L. reuteri* with selenium; OFT: open field test

To assess whether the treatments affected mice locomotor or exploratory activities, the number of crossings and rearings was evaluated in the OFT prior to the other behavioral tests. As shown in Fig. 2, the treatments did not produce significant differences in the number of crossings (*F*_(4,30)_ = 0.4563, *P* = 0.7670; Fig. 2A) or rearings (*F*_(4,30)_ = 0.4324, *P* = 0.7841; Fig. 2B) compared with the vehicle group (V), thereby excluding potential confounding effects or misleading results in the subsequent behavioral assessments.

### Effects of Lr, ILr, and ILr/Se on Depressive-Like Behaviors

**Fig. 3:**
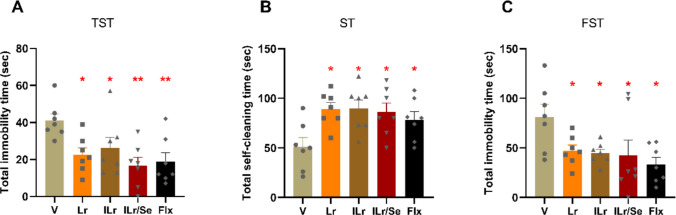
Effects of Lr, ILr, and ILr/Se in the tail suspension test (TST), splash test (ST), and forced swimming test (FST). **A** Immobility time in the TST. **B** Total self-cleaning time in the ST. **C** Immobility time in the FST. Data are expressed as mean ± standard error of the mean (SEM). *n* = 7 animals per group. * *P* < 0.05 and ** *P* < 0.01 vs. vehicle group. Data were analyzed using one-way analysis of variance (ANOVA) followed by the Newman–Keuls post hoc test. V: vehicle; Lr: *L. reuteri*; ILr: inactive *L. reuteri*; ILr/Se: inactive *L. reuteri* with selenium

Figure 3 illustrates the antidepressant-like effects of the Lr, ILr, and ILr/Se treatments in the TST, ST, and FST. Treatments with Lr, ILr, ILr/Se, and Flx significantly reduced immobility time in the TST compared with the vehicle group (*F*_(4,30)_ = 4.381, *P* = 0.0066; Fig. 3A). In the ST, all treatments increased total self-cleaning time relative to the vehicle group (*F*_(4,30)_ = 3.703, *P* = 0.0145; Fig. 3B). Similarly, in the FST, Lr, ILr, ILr/Se, and Flx treatments significantly decreased immobility time compared with the vehicle group (*F*_(4,30)_ = 3.346, *P* = 0.023; Fig. 3C).

### Effects of Lr, ILr, and ILr/Se on Anxiety-Like Behaviors

**Fig. 4:**
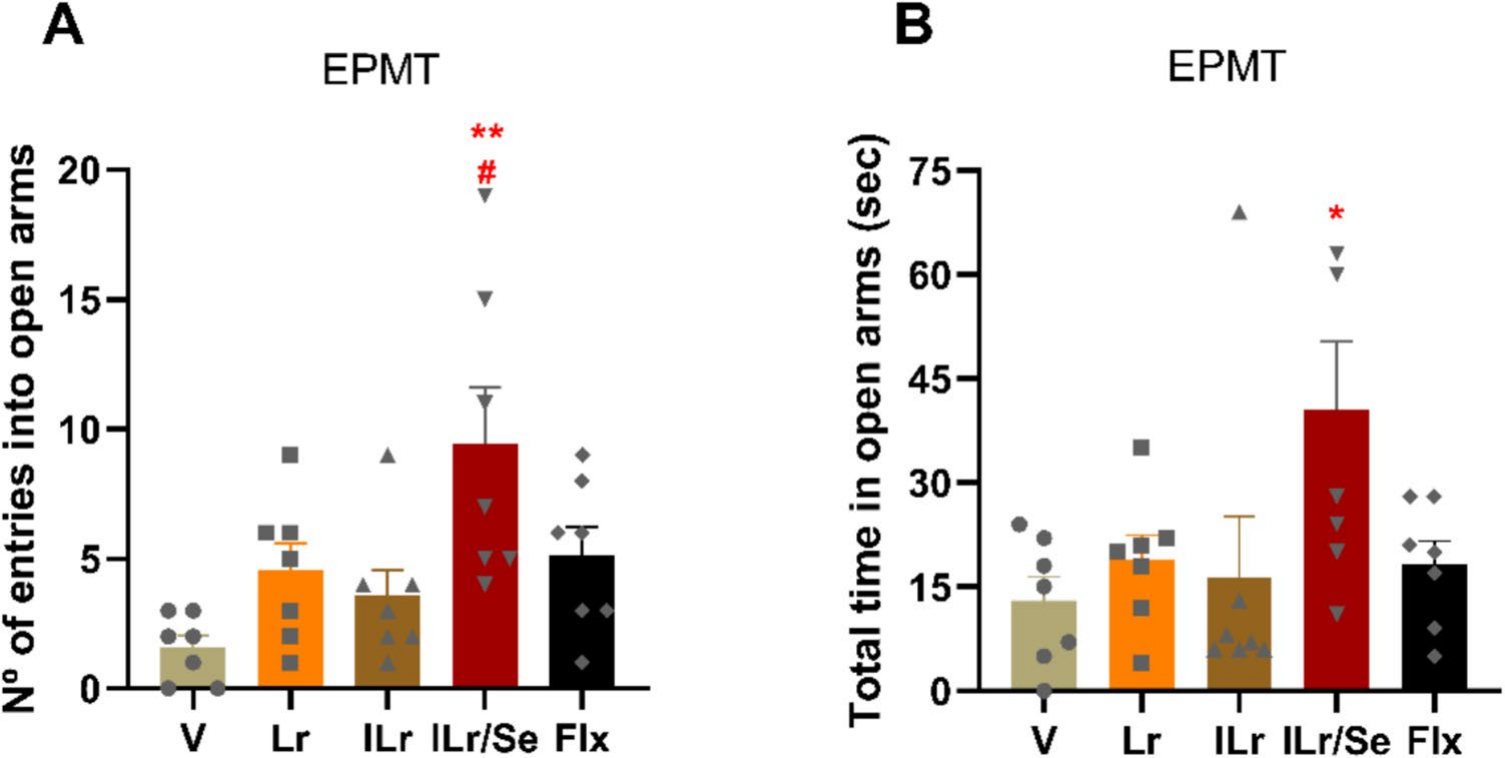
Effects of Lr, ILr, and ILr/Se in the elevated plus maze test (EPMT). **A** Number of entries into the open arms and **B** total time spent in the open arms. Data are expressed as mean ± standard error of the mean (SEM). * *P* < 0.05 and ** *P* < 0.01 compared with the vehicle group; and ^#^ *P* < 0.05 compared to the Lr and ILr groups. Data were analyzed using one-way analysis of variance (ANOVA) followed by the Newman–Keuls post hoc test. *n* = 7 animals per group. V: Vehicle; Lr: *L. reuteri*; ILr: inactive *L. reuteri*; ILr/Se: inactive *L. reuteri* with selenium

Figure 4 illustrates the anxiolytic-like effects of ILr/Se treatment in the EPMT. Treatment with ILr/Se significantly increased both the number of entries into the open arms (*F*_(4,30)_ = 5.060, *P* = 0.0031; Fig. 4A) and the time spent in the open arms compared with the vehicle group (*F*_(4,30)_ = 2.863, *P* = 0.0402; Fig. 4B). No significant differences were observed for the other treatments compared with the vehicle group.

### Lr, ILr, and ILr/Se Reduced Plasmatic Corticosterone Levels

**Fig. 5:**
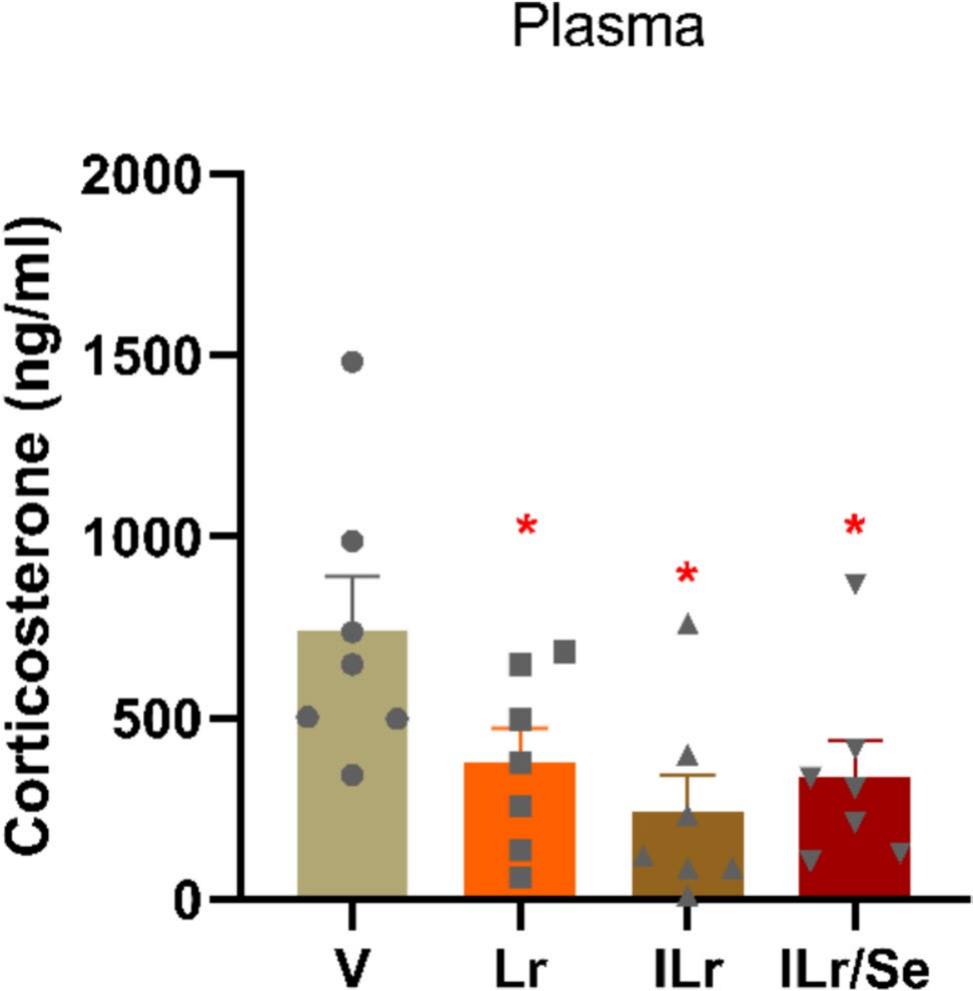
Effect of Lr, ILr, and ILr/Se on plasma corticosterone levels. Data are expressed as mean ± standard error of the mean (SEM). * *P* < 0.05 when compared with the vehicle group. Data were analyzed with a one-way analysis of variance (ANOVA) followed by Newman-Keuls post hoc test. *n* = 7 animals per group. V: Vehicle; Lr: *L. reuteri;* ILr: inactive *L. reuteri;* ILr/Se*:* inactive *L. reuteri* with selenium

As shown in Fig. 5, the treatments (Lr, ILr, and ILr/Se) significantly decreased plasma corticosterone levels in mice compared with the vehicle group (*F*_(3, 24)_ = 3.920; *P* = 0.0207).

### Lr, ILr, and ILr/Se Modulates Oxidative Stress Parameters

**Fig. 6:**
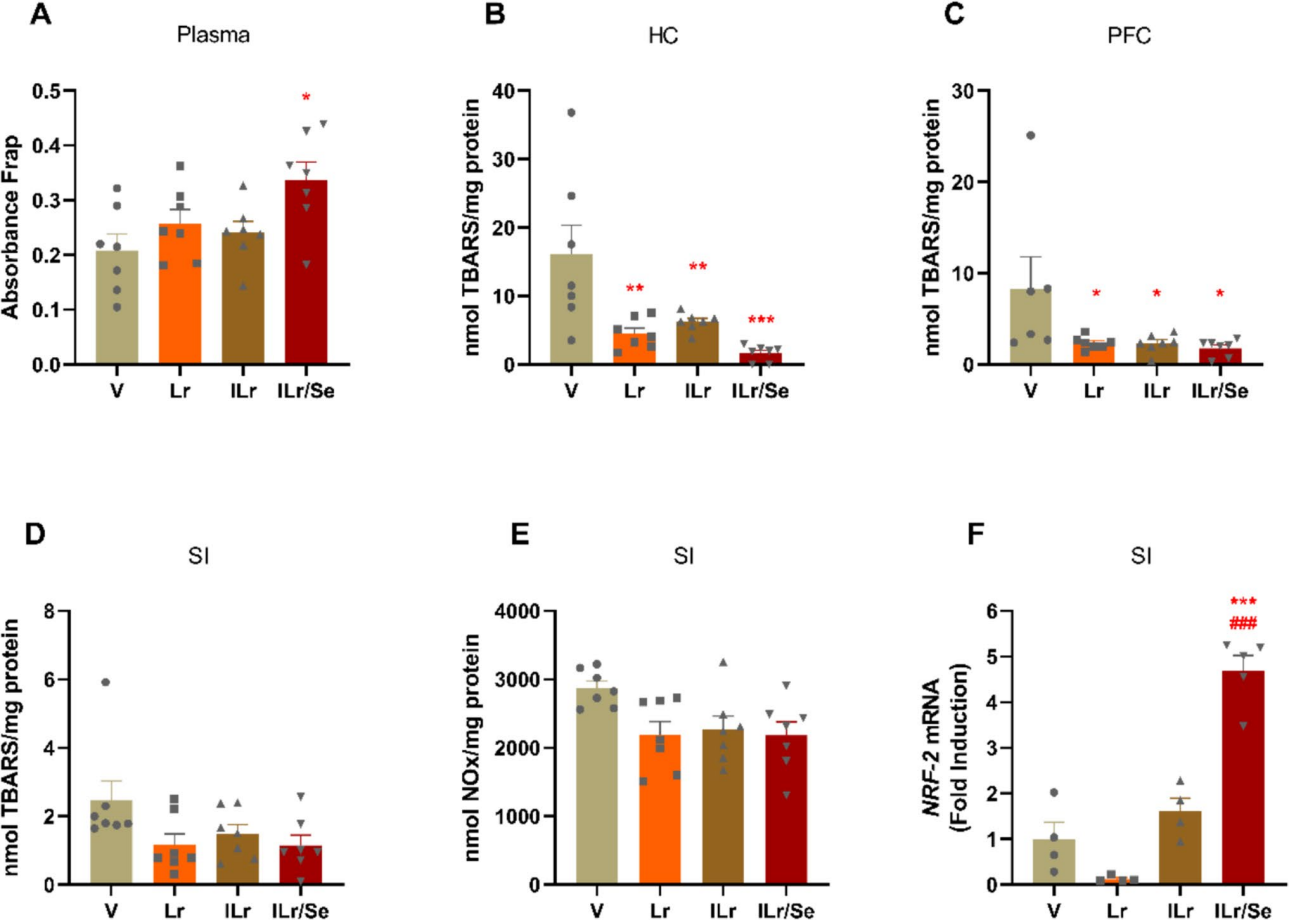
Effects of Lr, ILr, and ILr/Se on the ferric reducing/antioxidant power assay (FRAP) in the **A** plasma, on TBARS levels in the **B** hippocampus, **C** prefrontal cortex, and **D** small intestine, on NOx levels in the **E** small intestine and **F** *NRF-2* mRNA expression in the small intestine, determined by RT-qPCR using the 2^−ΔΔ*Ct*^ method and normalized to GAPDH. Data are expressed as mean ± standard error of the mean (SEM). * *P* < 0.05, ** *P* < 0.01, and *** *P* < 0.001 compared to the vehicle group; and ^###^ *P* < 0.001 compared to the Lr and ILr groups. *n* = 6–7 animals per group for FRAP, TBARS, and NOx data, and *n* = 4–5 for *NRF2* mRNA levels. Data were analyzed using a one-way analysis of variance (ANOVA) followed by Newman-Keuls post hoc test. V: Vehicle; Lr: *L. reuteri;* ILr: inactive *L. reuteri;* ILr/Se*:* inactive *L. reuteri* with selenium; HC: hippocampus; PCF: prefrontal cortex; SI: small intestine

Antioxidant capacity was assessed using the ferric reducing antioxidant power (FRAP) assay. We found that ILr/Se treatment significantly increased absorbance in the FRAP assay compared with the vehicle group (*F*_(3, 24)_ = 3.927; *P* = 0.0206, Fig. 6A), while other treatments showed no significant differences compared with the vehicle group. Additionally, the TBARS assay, a widely used method for evaluating lipid peroxidation in biological samples, revealed that Lr, ILr, and ILr/Se treatments reduced lipid peroxidation in the HC (*F*_(3, 24)_ = 7.988; *P* = 0.0007, Fig. 6B) and PFC (*F*_(3, 23)_ = 3.519; *P* = 0.0311) (Fig. 6C). However, no treatment significantly reduced lipid peroxidation in the SI, showing only a trend toward reduction (*F*_(3, 24)_ = 2.484; *P* = 0.0850, Fig. 6D) compared to the vehicle group. NOx levels were also measured in the SI, but no significant differences were observed among groups, with only a trend toward reduction observed (*F*_(3, 24)_ = 3.501; *P* = 0.0309, Fig. 6E).

The mRNA transcription of *NRF-2*, a key transcription factor in regulating antioxidant defense, cell metabolism, detoxification, and redox homeostasis, was analyzed. As shown in Fig. 6F, ILr/Se treatment significantly increased *NRF-2* mRNA transcription in the SI compared to the vehicle group (*F*_(3, 13)_ = 49.05; *P* < 0.0001), while other treatments showed no significant differences.

### Lr, ILr, and ILr/Se Modulates Inflammatory Markers

**Fig. 7:**
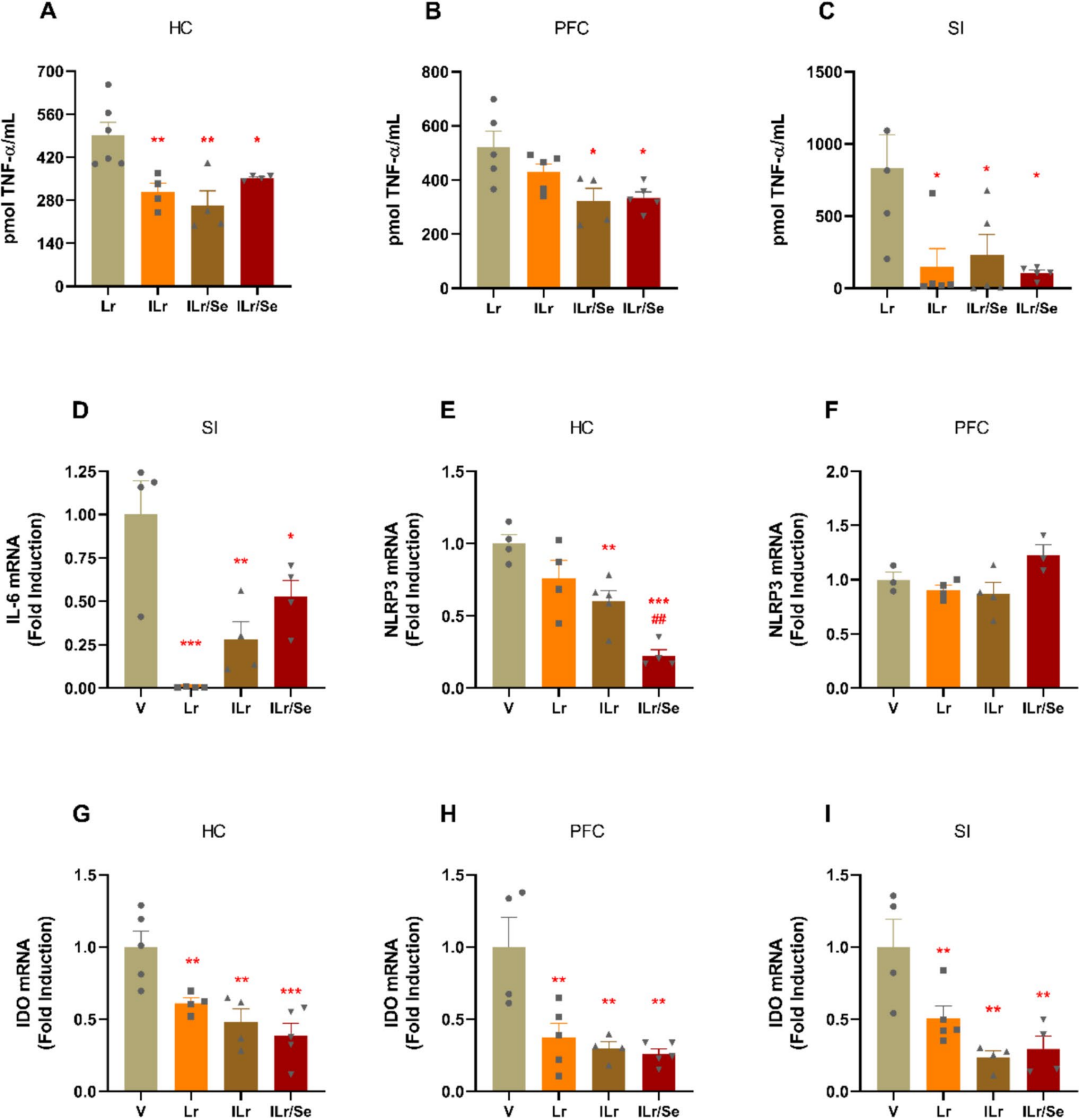
Effects of Lr, ILr, and ILr/Se on inflammatory parameters. TNF-α concentration in the hippocampus (**A**), prefrontal cortex (**B**), and small intestine (**C**), determined using a commercial ELISA kit. *IL-6* mRNA expression in the **D** small intestine*. NLRP3* mRNA expression in the hippocampus (**E**) and prefrontal cortex (**F**). *IDO* mRNA expression in the hippocampus (**G**), prefrontal cortex (**H**), and small intestine (**I**). Relative mRNA expression was determined by RT-qPCR using the 2^−ΔΔ*Ct*^ method and normalized to GAPDH. Data are expressed as mean ± standard error of the mean (SEM). * *P* < 0.05, ** *P* < 0.01, and *** *P* < 0.001 compared with the vehicle group; ^##^ *P* < 0.01 compared with the Lr and ILr groups. Data were analyzed with a one-way analysis of variance (ANOVA) followed by Newman-Keuls post hoc test. *n* = 4–6 animals per group for TNF-α levels, and *n* = 3–5 animals for *NLRP3*, *IDO* and *IL-6* mRNA levels. V: vehicle; Lr: *L. reuteri;* ILr: inactive *L. reuteri;* ILr/Se*:* inactive *L. reuteri* with selenium; HC: hippocampus; PCF: prefrontal cortex; SI: small intestine

Our results demonstrate that the treatment with Lr, ILr, and ILr/Se reduced TNF-α levels in HC compared to the vehicle group (*F*_(3, 14)_ = 7.590; *P* = 0.0030, Fig. 7A). Similarly, the treatment with ILr and ILr/Se decreased the TNF-α levels in PFC compared to the vehicle group, but the Lr treatment was not significantly different (*F*_(3, 15)_ = 4.898; *P* = 0.014, Fig. 7B). All treatments decreased the TNF-α levels in SI compared to the vehicle group (*F*_(3, 16)_ = 5.180; *P* = 0.0108, Fig. 7C). In the SI, mRNA levels of IL-6 were significantly lower in the Lr, ILr, and ILr/Se groups compared to the vehicle group (*F*_(3, 12)_ = 12.22; *P* = 0.0006, Fig. 7D). Figure 7E shows that ILr and ILr/Se treatments significantly reduced *NLRP3* mRNA expression in the HC compared to the vehicle group (*F*_(3, 13)_ = 15.09; *P* = 0.0002), whereas Lr treatment did not show a statistically significant difference. No treatment affected *NLRP3* mRNA expression in the PFC compared to the vehicle group (*F*_(3, 10)_ = 3.524; *P* = 0.0566, Fig. 7F). Figure 7G–I demonstrates that all treatments reduced *IDO* mRNA expression in the HC (*F*_(3, 14)_ = 9.887; *P* = 0.0009), PFC (*F*_(3, 14)_ = 9.404; *P* = 0.0012), and SI (*F*_(3, 13)_ = 8.810; *P* = 0.0019), respectively, compared to the vehicle group.

### Lr, ILr, and ILr/Se Modulate Neuroplasticity Parameters

**Fig. 8:**
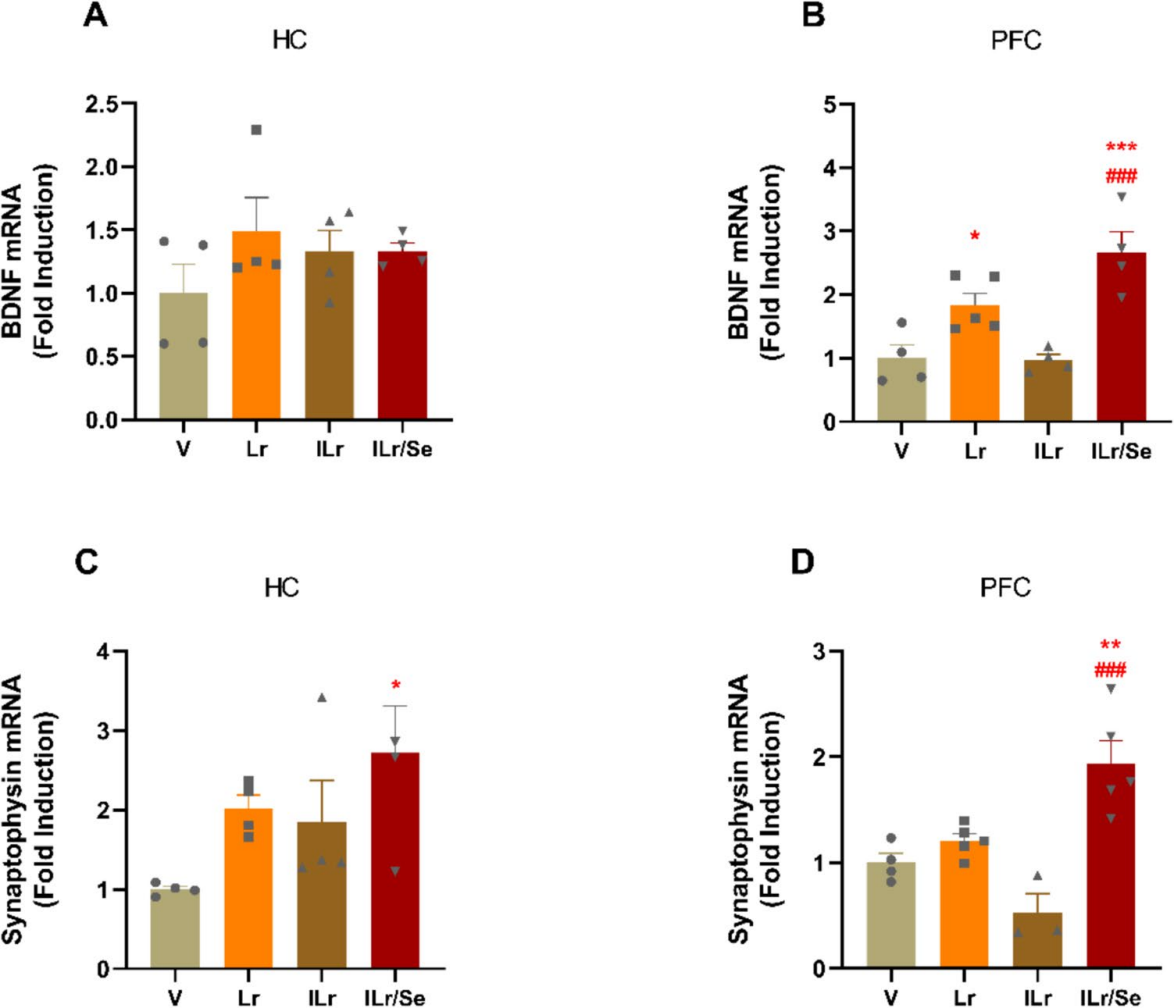
Effects of Lr, ILr, and ILr/Se on neuroplasticity. BDNF mRNA expression in the hippocampus (**A**) and prefrontal cortex (**B**). S*ynaptophysin* mRNA expression in the hippocampus (**C**) and prefrontal cortex (**D**). Relative mRNA expression was determined by RT-qPCR using the 2^−ΔΔ*Ct*^ method and normalized to GAPDH. Data are expressed as mean ± standard error of the mean (SEM). * *P* < 0.05 and ** *P* < 0.01 compared with the vehicle group; ^###^ *P* < 0.001 compared with the Lr and ILr groups. Data were analyzed with a one-way analysis of variance (ANOVA) followed by Newman-Keuls post hoc test. *n* = 4–5 animals per group. V: vehicle; Lr: *L. reuteri;* ILr: inactive *L. reuteri;* ILr/Se*:* inactive *L. reuteri* with selenium; HC: hippocampus; PFC: prefrontal cortex; SI: small intestine

As shown in Fig. 8A, none of the treatments affected *BDNF* mRNA expression in the HC compared with the vehicle group (*F*_(3, 12)_ = 1.102; *P* = 0.3863). However, *BDNF* mRNA expression was increased in the PFC following Lr and ILr/Se treatments (*F*_(3, 13)_ = 12.78; *P* = 0.0004, Fig. 8B). Additionally, ILr/Se treatment significantly increased *synaptophysin* mRNA expression in the HC (*F*_(3, 12)_ = 3.685; *P* = 0.0433, Fig. 8C) and PFC (*F*_(3, 13)_ = 13.96; *P* = 0.0002, Fig. 8D) compared with the vehicle group, while other treatments showed no significant differences in either brain region.

### ILr/Se Modulates the PI3k/Akt/mTOR Pathway

**Fig. 9:**
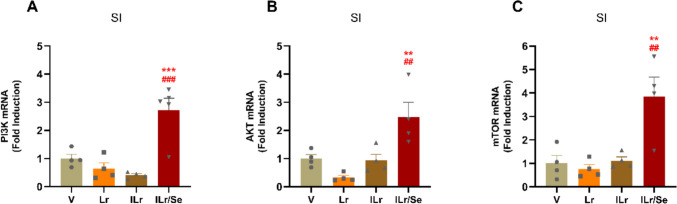
Effects of Lr, ILr, and ILr/Se on PI3k/Akt/mTOR pathway. *PI3K* mRNA expression in the small intestine (**A**). *AKT* mRNA expression in the small intestine (**B**). *mTOR* mRNA expression in the small intestine (**C**). Relative mRNA expression was determined by RT-qPCR using the 2^−ΔΔ*Ct*^ method and normalized to GAPDH. Data are expressed as mean ± standard error of the mean (SEM). ** *P* < 0.01 and *** *P* < 0.001 compared with the vehicle group; ^##^ *P* < 0.01 and ^###^ *P* < 0.001 compared with the Lr and ILr groups. Data was analyzed with a one-way analysis of variance (ANOVA) followed by Newman-Keuls. *n* = 4–5 animals per group. V: vehicle; Lr: *L. reuteri;* ILr: inactive *L. reuteri;* ILs/Se: inactive *L. reuteri* with selenium; SI: small intestine

Figure 9A–C shows that ILr/Se treatment significantly increased *PI3K, AKT,* and *mTOR* mRNA fold transcription in the SI compared with the vehicle group (*F*_(3, 13)_ = 14,87; *P* = 0.0002, *F*_(3, 12)_ = 9.247; *P* = 0.0019, *F*_(3, 12)_ = 9.534; *P* = 0.0017, respectively). In contrast, Lr and ILr treatments did not exhibit statistically significant differences compared with the vehicle group.

## Discussion

Our findings demonstrate that treatment with *Lactobacillus reuteri*, whether in its viable form (Lr), its inactivated postbiotic form (ILr), or its selenium-enriched variant (ILr/Se), induced antidepressant-like effects in mice [47]. Importantly, both postbiotic preparations produced biological responses comparable to the live strain, underscoring the therapeutic relevance of non-viable microbial derivatives. Notably, the ILr/Se treatment also produced a robust anxiolytic-like effect. All treatments were associated with reductions in corticosterone levels, modulation of the antioxidant activity, key inflammatory and neuroplasticity-related markers, underscoring the relevance of microbiota–gut–brain interactions in mood regulation. To the best of our knowledge, this is the first study to evaluate the behavioral and biological impact of a medium containing *L. reuteri* LRE02 in both its inactivated and selenium-enriched forms. The novelty of this work lies in demonstrating the therapeutic promise of these two postbiotic strategies, thereby broadening the conceptual and practical landscape of postbiotic applications in mental health. Moreover, postbiotics offer practical advantages, including greater stability, safety, and reproducibility, while retaining the capacity to modulate gut–immune communication, positioning them as viable alternatives or complements to traditional therapies [2–4].

The antidepressant-like effects observed in both the TST and FST were evident across all treatment groups, Lr, ILr, and ILr/Se, with each significantly reducing immobility time of the animals. Our findings align with those of Saleh et al. (2024) [48], who reported enhanced mobility in the TST following a 2-week treatment with *L. reuteri* in mice exposed to early-life stress. Likewise, a recent study using fermented rice supplemented with *L. reuteri* also demonstrated reduced immobility in the FST in a stress-induced depression model [49]. However, the most compelling aspect of our findings lies in the performance of the two inactivated preparations. This highlights the therapeutic relevance of microbial structural components and metabolites preserved after inactivation, which may modulate gut-derived signaling pathways capable of influencing central neurotransmission [24]. Collectively, our data highlights not only the efficacy of the live microorganism but, importantly, the promising therapeutic potential of its inactivated forms.

Stress is a well-established risk factor for the development of mental disorders [50]. Importantly, widely used behavioral assays such as the TST and the FST are themselves acute stressors. By exposing animals to an inescapable stressful situation, these tests reliably activate neuroendocrine pathways associated with depressive-like behavior and are therefore used as screening tools to identify potential antidepressant therapies. In response to such acute stress, catecholamines are released, initiating the classical “fight-or-flight” response, which subsequently triggers activation of the hypothalamic–pituitary–adrenal (HPA) axis and increases glucocorticoid secretion [51]. Corticosterone, the rodent equivalent of human cortisol, is thus a key biomarker of stress-induced HPA axis activation. In our study, we observed a reduction in corticosterone levels with the treatments with *L. reuteri*. These results are consistent with those reported by Saleh et al. (2024) [48] and Tyagi et al. (2025) [49], who similarly showed that *L. reuteri* mitigates HPA axis overactivation in stressed mice. Collectively, these findings support the idea that both probiotic and postbiotic approaches can modulate the neuroendocrine stress response, ultimately reduce glucocorticoid secretion and improve behavioral outcomes [21, 22].

A key finding of this study was the robust anxiolytic-like effect elicited by ILr/Se in the EPMT, reflected by a marked increase in both open-arm entries and time spent in the open arms. This result strongly suggests that selenium enrichment amplifies the anxiolytic potential of *L. reuteri*–derived postbiotics, likely through combined actions on oxidative and inflammatory pathways, two biological processes tightly linked to anxiety. Supporting this hypothesis, ILr/Se treatment significantly upregulated NRF-2 mRNA expression in the SI, indicating activation of endogenous antioxidant defenses. Selenium is an essential cofactor for selenoproteins involved in redox regulation, and *L. reuteri* strains are known to biotransform inorganic selenium (e.g., sodium selenite) into organic forms such as selenocysteine and selenomethionine, which are less toxic and more bioactive. Although the present study did not directly assess this conversion, the viability of the selenium-exposed strain for several hours suggests that such biotransformation may have occurred and contributed to the pronounced behavioral effects observed in the ILr/Se group. It should be noted that fluoxetine, used as a positive control to validate behavioral assays, exhibited antidepressant-like effects in depression-related tests but did not produce significant anxiolytic effects. This discrepancy may be attributed to the relatively low dosage administered.

Biochemical analyses revealed that all three treatments: Lr, ILr, and ILr/Se were effective in reducing lipid peroxidation in both the PFC and HC, highlighting a shared antioxidant and neuroprotective potential. Among these, ILr/Se exhibited additional effects at the systemic and intestinal levels: this treatment uniquely reduced plasma FRAP values and significantly increased NRF2 mRNA expression in the SI. These findings may be partly explained by the presence of selenium, which can enhance antioxidant defenses by supporting selenoproteins and redox-active enzymes, contributing to NRF2 pathway activation and improved systemic oxidative control. Our results are consistent with reports showing that *L. reuteri* can decrease malondialdehyde (MDA) levels in models of neuroinflammation [52], and with clinical evidence linking probiotic supplementation to reductions in oxidative damage markers such as MDA [53]. Additionally, proteomic data demonstrates that *L. reuteri* upregulates selenium-related oxidoreductases under sodium selenite exposure [54] further supporting the enhanced redox effects observed in ILr/Se. Importantly, the fact that ILr and ILr/Se, both inactivated preparations, produced meaningful biochemical benefits reinforces the value of *L. reuteri* as a postbiotic. Postbiotics offer advantages such as greater stability, safety, and shelf-life when compared to live strains, while still retaining bioactive components capable of modulating oxidative stress. Together, these findings suggest that all treatments confer antioxidant protection, with ILr/Se providing complementary mechanisms related to selenium-driven redox modulation.

Inflammatory modulation was another central outcome of this study. All treatments (Lr, ILr, and ILr/Se) significantly reduced TNF-α levels in the hippocampus, while ILr and ILr/Se also decreased TNF-α in the prefrontal cortex. In the small intestine, all three interventions lowered TNF-α expression, indicating systemic anti-inflammatory activity. These effects are relevant given the strong link between elevated pro-inflammatory cytokines and psychiatric disorders [58]. *L. reuteri* is known to modulate inflammation through multiple mechanisms; notably, Thomas et al. (2012) [11] demonstrated that the strain converts L-histidine into histamine, a metabolite capable of suppressing TNF-α production. Consistent with these findings, daily intake of a probiotic blend containing *L. reuteri* has been shown to improve oxidative status and decrease inflammatory markers, including TNF-α [7]. In addition, our treatments, whether live or inactivated, downregulated IL-6 expression in the intestine, a cytokine commonly elevated in major depressive disorder [55, 56]. Similar reductions in IL-6 have been reported in studies evaluating *L. reuteri* strains in models of depression and intestinal inflammation [21], further supporting the anti-inflammatory profile observed here.

Treatments with ILr and ILr/Se also significantly downregulated NLRP3 mRNA expression in the hippocampus, suggesting inhibition of inflammasome activation, a pathway strongly linked to neuroinflammation, depression, and anxiety [57]. No changes were observed in the PFC, indicating that there are region-specific differences in inflammatory responsiveness. The IDO–kynurenine pathway and the NLRP3 inflammasome are widely recognized for their roles in inflammation and mood disorders [58]. IDO catalyzes the degradation of tryptophan toward kynurenine, promoting the production of pro-inflammatory cytokines and reducing substrate availability for serotonin synthesis [59–61]. Because nearly 90% of serotonin is produced in the gut, maintaining adequate tryptophan levels is essential for intestinal and central nervous system function [62]. Excessive IDO activity, therefore, contributes to decreased serotonin and increased vulnerability to mood disturbances. In this study, all treatments downregulated IDO expression in both the hippocampus and PFC. Importantly, they also normalized IDO mRNA levels in the small intestine, suggesting a protective effect on gut-derived serotonin production. These findings reinforce the role of *L. reuteri*, whether live or inactivated, in modulating key inflammatory and metabolic pathways within the microbiota-gut-brain axis.

Our findings also point to a meaningful interaction between ILr/Se treatment and the PI3K/Akt/mTOR signaling pathway in the SI. This intracellular cascade governs essential processes such as cell survival, metabolism, and stress adaptation [63], and has been increasingly implicated in neuroprotection and mood regulation. In our study, ILr/Se significantly upregulated PI3K, AKT, and mTOR mRNA expression in the intestine, suggesting that selenium-enriched postbiotic supplementation can modulate pathways closely linked to neuronal resilience and synaptic plasticity. These results align with emerging evidence showing that gut microbes and their metabolites can influence PI3K/Akt/mTOR signaling, thereby shaping both intestinal and brain physiology [7, 64]. The activation of this pathway may underlie the behavioral and biochemical improvements observed in the ILr/Se group, offering a plausible mechanistic link between gut-level modulation, reduced stress reactivity, and the resulting antidepressant- and anxiolytic-like effects. The increased expression of NRF2 further supports this interpretation, as this transcription factor orchestrates endogenous antioxidant defenses by regulating genes involved in cellular protection against oxidative stress. In line with this, the enhanced total antioxidant capacity detected in the FRAP assay reflects a greater tissue reducing potential. Likewise, the reduced TBARS levels indicate lower lipid peroxidation, suggesting diminished oxidative damage to cellular membranes. Taken together, these findings point to a scenario in which modulation of the PI3K/Akt/mTOR signaling pathway is closely associated with antioxidant and cytoprotective mechanisms that likely contribute to the observed behavioral outcomes.

Beyond intracellular signaling, ILr/Se treatment also influenced key neuroplasticity markers. Neuroplasticity is strongly influenced by HPA axis dysregulation, alterations in neurotrophic factors, and inflammatory signaling, processes closely linked to the development of depression [65]. In this study, ILr/Se significantly increased synaptophysin mRNA expression in both the HC and PFC, indicating improved synaptic integrity and connectivity. This result is consistent with evidence associating synaptophysin upregulation with antidepressant- and anxiolytic-like outcomes through enhanced neurotransmission [57]. ILr/Se also elevated BDNF expression in the PFC, reinforcing its role in promoting neuronal survival and synaptic remodeling. Although the other treatments did not significantly affect BDNF levels in the HC, their behavioral and biochemical effects still support a beneficial impact on neuroplasticity. The observed neurotrophic responses may be partially mediated by SCFAs, microbial metabolites known to influence BDNF expression [49]. *L. reuteri* is a recognized SCFA producer [63, 66], which strengthens the hypothesis that both live and inactivated forms can modulate neuroplasticity through gut–brain axis mechanisms. Together, these findings highlight the capacity of *L. reuteri*, particularly in its selenium-enriched postbiotic form, to support synaptic resilience and contribute to the antidepressant- and anxiolytic-like effects observed in this study.

This study provides foundational insights into the potential of selenium-enriched Lactobacillus reuteri postbiotics. While our results offer robust evidence of the formulation’s bioactivity, certain aspects warrant consideration for future research. The current study focused on comparing the effects of the postbiotic matrix with and without selenium; thus, including an inorganic selenium-only control in future investigations would be valuable for further delineating the specific contribution of the mineral. Similarly, while our molecular data offer strong evidence of pathway activation, extending these findings to protein-level analyses in subsequent studies will provide a more comprehensive mechanistic view. Furthermore, this initial screening was conducted using acute paradigms in healthy male mice; broadening the scope in future studies to include disease models, female animals, and analysis of gut microbiota composition will be essential to fully elucidate the translational potential and specific biological mediators of these effects. Regarding the formulation, future characterization of selenium speciation will also be beneficial to optimize our understanding of the bio-transformative processes involved.

In conclusion, our study provides robust evidence that both live and inactivated forms of *L. reuteri* exert significant antidepressant- and anxiolytic-like effects, underscoring the therapeutic promise of postbiotics as stable, safe, and biologically active interventions. The inactivated strain consistently influenced corticosterone levels, neuroinflammatory markers, redox balance, and indicators of neuroplasticity. Selenium enrichment further enhanced several of these effects, most notably anxiolytic-like behavior, antioxidant responses, and activation of the PI3K/Akt/mTOR pathway, demonstrating that strategic postbiotic fortification may potentiate bioactivity. Together, these findings advance the growing field of microbiota-based mental health interventions and highlight postbiotics, especially inactivated *L. reuteri* formulations, as promising candidates for future neuropsychiatric therapies. Nonetheless, as noted above, these results should be interpreted with caution, given the reliance on acute behavioral assays in healthy animals to screen for antidepressant‑ and anxiolytic‑like effects, as well as the absence of direct microbiota analyses. Additional studies are required to deepen our understanding of the underlying molecular pathways and evaluate the effects of selenium‑enriched *L. reuteri* in induced animal models of depression and anxiety.

## Data Availability

Data will be made available on request.
